# Metastatic pancreatic neuroendocrine tumors feature elevated T cell infiltration

**DOI:** 10.1172/jci.insight.160130

**Published:** 2022-12-08

**Authors:** Jacques Greenberg, Jessica Limberg, Akanksha Verma, David Kim, Xiang Chen, Yeon J. Lee, Maureen D. Moore, Timothy M. Ullmann, Jessica W. Thiesmeyer, Zachary Loewenstein, Kevin J. Chen, Caitlin E. Egan, Dessislava Stefanova, Rohan Bareja, Rasa Zarnegar, Brendan M. Finnerty, Theresa Scognamiglio, Yi-Chieh Nancy Du, Olivier Elemento, Thomas J. Fahey, Irene M. Min

**Affiliations:** 1 Department of Surgery,; 2 Caryl and Israel Englander Institute for Precision Medicine, Institute for Computational Biomedicine, and; 3 Department of Pathology and Laboratory Medicine, Weill Cornell Medicine, New York, New York, USA.

**Keywords:** Immunology, Therapeutics, Cancer, Cancer immunotherapy, Cellular immune response

## Abstract

Pancreatic neuroendocrine tumors (PNETs) are malignancies arising from the islets of Langerhans. Therapeutic options are limited for the over 50% of patients who present with metastatic disease. We aimed to identify mechanisms to remodel the PNET tumor microenvironment (TME) to ultimately enhance susceptibility to immunotherapy. The TMEs of localized and metastatic PNETs were investigated using an approach that combines RNA-Seq, cancer and T cell profiling, and pharmacologic perturbations. RNA-Seq analysis indicated that the primary tumors of metastatic PNETs showed significant activation of inflammatory and immune-related pathways. We determined that metastatic PNETs featured increased numbers of tumor-infiltrating T cells compared with localized tumors. T cells isolated from both localized and metastatic PNETs showed evidence of recruitment and antigen-dependent activation, suggestive of an immune-permissive microenvironment. A computational analysis suggested that vorinostat, a histone deacetylase inhibitor, may perturb the transcriptomic signature of metastatic PNETs. Treatment of PNET cell lines with vorinostat increased chemokine CCR5 expression by NF-κB activation. Vorinostat treatment of patient-derived metastatic PNET tissues augmented recruitment of autologous T cells, and this augmentation was substantiated in a mouse model of PNET. Pharmacologic induction of chemokine expression may represent a promising approach for enhancing the immunogenicity of metastatic PNET TMEs.

## Graphical abstract

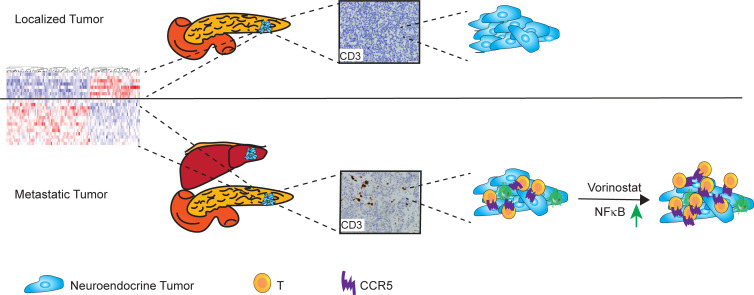

## Introduction

Neuroendocrine tumors (NETs) are rare, but their incidence has been steadily increasing over the last 50 years (1). NETs can arise from cells of the endocrine and nervous systems. Pancreatic NETs (PNETs) occur in approximately 0.8 of 100,000 individuals in the United States, comprising 2%–7% of all pancreatic neoplasms (2–4). Most PNETs are classified as nonfunctional (nonhormone secreting), and their disease progression is prolonged; however, the prognosis for patients with metastatic PNETs is poor (5, 6). Localized PNETs are associated with a 5-year overall survival of 90%–93%, whereas regional lymph node metastasis or distant metastasis reduce the 5-year survival to 60%–77% or 20%–40%, respectively (1–3, 7). Unfortunately, approximately 50%–80% of patients with PNETs either have lymph node or distant metastasis, usually involving the liver, at the time of diagnosis (1, 8).

Surgical resection serves as the mainstay of treatment for the majority of PNETs (2, 9). Despite surgery, patients with metastases often suffer from recurrent or progressive disease (10). Unfortunately, the efficacy of therapeutic options for adjuvant treatment or treatment of metastatic disease is limited (2, 10–12). Systemic treatment options include a combination of chemotherapeutic drugs and targeted inhibitors of the MTOR pathway (everolimus), or tyrosine kinase inhibitors (sunitinib) (2, 13–15). Somatostatin analogues and liver-directed interventional therapies in unresectable disease have also demonstrated some clinical benefit (2, 16, 17). However, each of these treatments provides limited benefit; sunitinib only prolongs median progression-free survival by 4 months, while liver-directed radioembolization prolongs overall survival by 6 months (15, 17).

Within the last decade, immunotherapy targeting immune checkpoint molecules has emerged as a highly effective treatment option in a subset of patients across a range of immunogenic cancers with elevated baseline T cell infiltration, including melanomas and non–small cell lung cancers, or in cancers with mismatch repair deficiencies (18–23). These therapies have helped usher in an era whereby modulation of tumor microenvironments (TME) represents a promising therapeutic avenue (24, 25). However, early clinical data with respect to immune checkpoint blockade has been less promising in patients with PNETs (6, 26, 27). This has been attributed to a number of factors: a low rate of tumor mutational burden, generally inadequate T cell infiltration, and relatively low expression of immune regulation markers, including programmed cell death 1 (PDCD1) and its ligands (PD-L1 and PD-L2) (18, 25, 28, 29). The wide variety of morphologies, behaviors, and genetic heterogeneity observed among PNETs has contributed to inconsistent reports of prognostic association for each of these markers (25, 30). For example, the degree of tumor-infiltrating lymphocytes (TIL) in PNETs has been variably related to overall prognosis (25, 29, 30). Together, these studies highlight the complexity of the TME in PNETs, but they do not specifically address the interplay of these components in the progression of localized to metastatic disease.

To investigate early changes accompanying disease progression to metastasis, we performed RNA-Seq analysis on surgically excised primary tumors originating from both localized and metastatic PNETs. We aimed to determine whether unique molecular signatures distinguished metastatic PNETs from localized tumors in the primary tumor site in the pancreas. This information was then used to define characteristics associated with tumor and immune cell interaction, depending upon the metastatic potential, followed by a comprehensive analysis of patients’ tumor tissues using IHC and flow cytometry. We identified potential therapeutic options for PNET management and delineated the mechanism by which increased T cell recruitment to the tumor could be optimized. This finding was also supported when tested in a mouse model developing insulin-secreting PNETs.

## Results

### RNA-Seq identifies distinct gene expression profiles unique to localized and metastatic PNETs.

**Figure 1:**
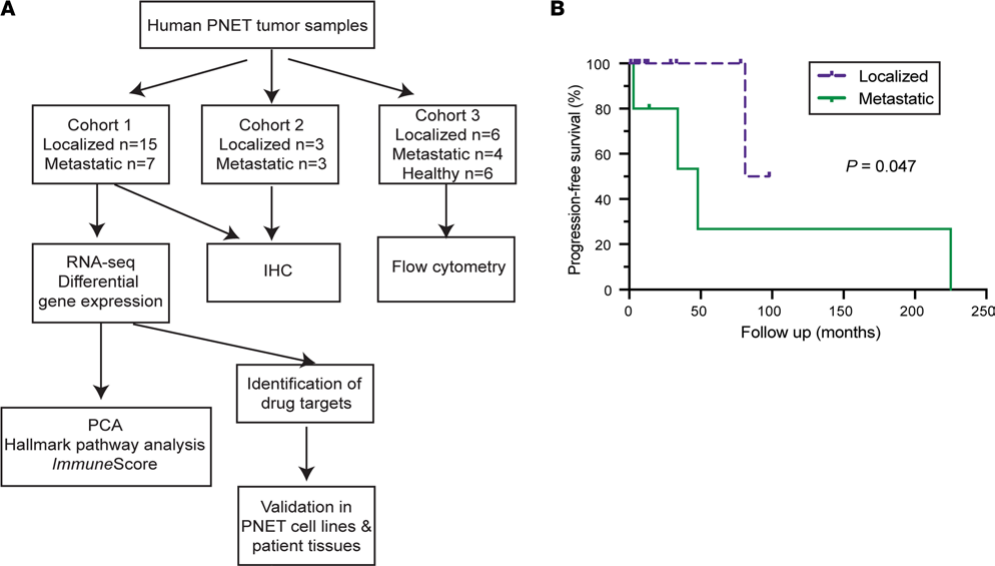
Schematic diagram of bioinformatic analysis of this study. (**A**) Tissue was collected from the primary tumors of 22 patients with PNETs for RNA-Seq in Cohort 1. Gene expression was compared between metastatic and localized tumors to derive a metastatic expression profile. Principal component analysis (PCA), Hallmark pathway, and ESTIMATE *Immune*Score analyses were performed. A list of therapeutic drugs was generated based on the differential expression profiles between metastatic and localized tumors, and a candidate drug was examined for its effect on PNET cell lines and patient tissues. IHC was performed in 12 patients from Cohort 1 along with Cohort 2. Flow cytometry analysis in Cohort 3 was performed with additional tumors and blood samples. (**B**) Progression-free survival curves generated for patients with metastatic and localized disease (Cohort 1 in **A**). Progression-free survival was greater for patients with localized PNETs compared with those with metastatic PNETs (*P* = 0.047, Gehan-Breslow-Wilcoxon test). The median progression-free survival for those with localized disease was 89.5 months, whereas it was 48 months for metastatic disease.

To determine whether transcriptional changes manifest between localized and metastatic PNETs, we performed RNA-Seq analysis on primary tumor tissue of each tumor type. A schematic diagram of the sample collection and bioinformatic analysis performed in this study is outlined in Figure 1A. RNA was extracted from primary tumor tissue in 22 patients — 15 with localized tumors and 7 with metastatic disease, defined as those with spread to lymph nodes or liver (Table 1 and Supplemental Table 1; supplemental material available online with this article; https://doi.org/10.1172/jci.insight.160130DS1). There were no statistically significant differences in patient age, sex, tumor location in the pancreas, or tumor functionality between the localized and metastatic groups. Tumor sizes were marginally different between these 2 groups — metastatic tumors were larger, although this did not reach statistical significance, likely due to the small sample size. Inherently, those with metastatic disease represented more advanced tumor stages than those with localized disease (*P* = 0.018). Two grade 3 primary lesions were included in the metastatic group, and the rest of the tumors were categorized as low grade (G1) and intermediate grade (G2) according to the World Health Organization (WHO) 2017 classification. Although the majority of tumors were nonfunctional (63%, *n* = 14), 8 patients had functional tumors (insulinomas [*n* = 7] and vasoactive intestinal peptide tumor [VIPoma, *n* = 1]). Two patients had isolated nodal disease, 3 patients had nodal and liver disease, and 2 patients had isolated liver disease. Patients with localized disease had a longer progression-free survival than those with metastatic disease (89.5 versus 48.0 months, *P* = 0.047), with the only death occurring in the metastatic cohort (Figure 1B).

**Figure 2:**
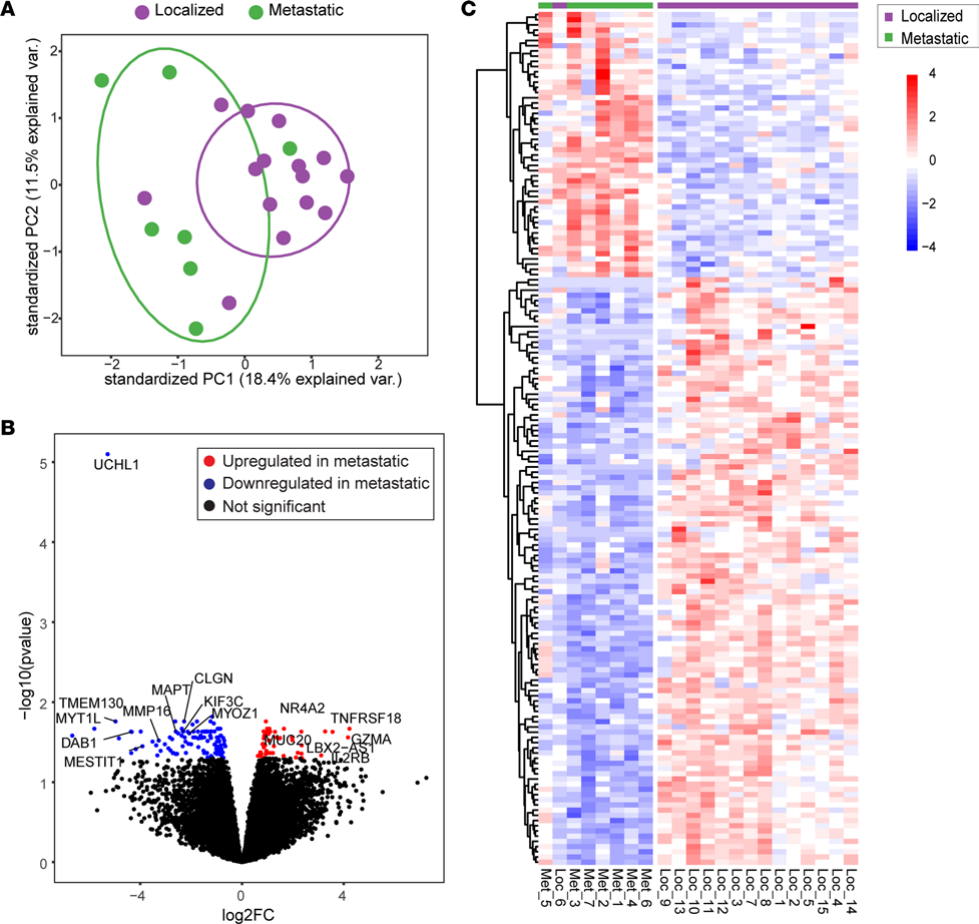
Unsupervised hierarchical clustering identifies differential expression between localized and metastatic PNETs. (**A**) PCA of RNA-Seq in PNET tumors of Cohort 1. (**B**) Volcano plot shows log_2_ fold change plotted against significance (–log_10_ *P* scale, Wald’s test). Each dot represents an individual gene differentially expressed (FDR < 0.05). Positive and negative log_2_ changes indicate enrichment and downregulation in metastatic tumors, respectively. (**C**) Unsupervised hierarchical clustering of RNA signature using log-normalized FPKM.

Principal component analysis (PCA) of the RNA-Seq expression profiles revealed a pronounced clustering of tumors according to their metastatic potential, with a greater degree of variance among metastatic compared with localized tumors (Figure 2A). PNETs represent a heterogenous group of neoplasms with diverse genomic signatures (2); as a result, the relatively small percentage of variance of the first 2 principal components identified in our data set may have arisen due to the fact that the variation in our data stems from many factors, with each contributing a relatively small part of the total variation instead of a few factors accounting for the majority of the variation. Nevertheless, the distinguishable clustering of tumors according to their metastatic status appeared robust, except for 3 tumors. These included 2 localized tumor samples clustered in the metastatic tumor group. One was an intermediate grade tumor with 10% Ki67 and perineural invasion, which developed hepatic metastasis 47 months postoperatively. The other tumor was low grade and among the largest localized tumors in the cohort (4 cm with 16% Ki67). On the other hand, the 1 metastatic tumor that grouped with the localized tumors in the PCA was low grade and had the lowest mitotic count (<2 of 10) but with < 3% Ki67.

Pairwise differential gene-expression analysis identified 165 differentially expressed genes between metastatic and localized PNETs (FDR < 0.05 and fold-change > 1.5) (Figure 2B). Fifty-two genes were upregulated in metastatic compared with localized PNETs, whereas 113 were downregulated. A heatmap display of unsupervised hierarchical clustering analysis revealed that metastatic and localized PNETs were largely separated into 2 groups based upon contributions from varying levels of mRNA expression changes (Figure 2C).

### Enrichment for immune-related genes in primary tissue of metastatic PNETs.

**Figure 3:**
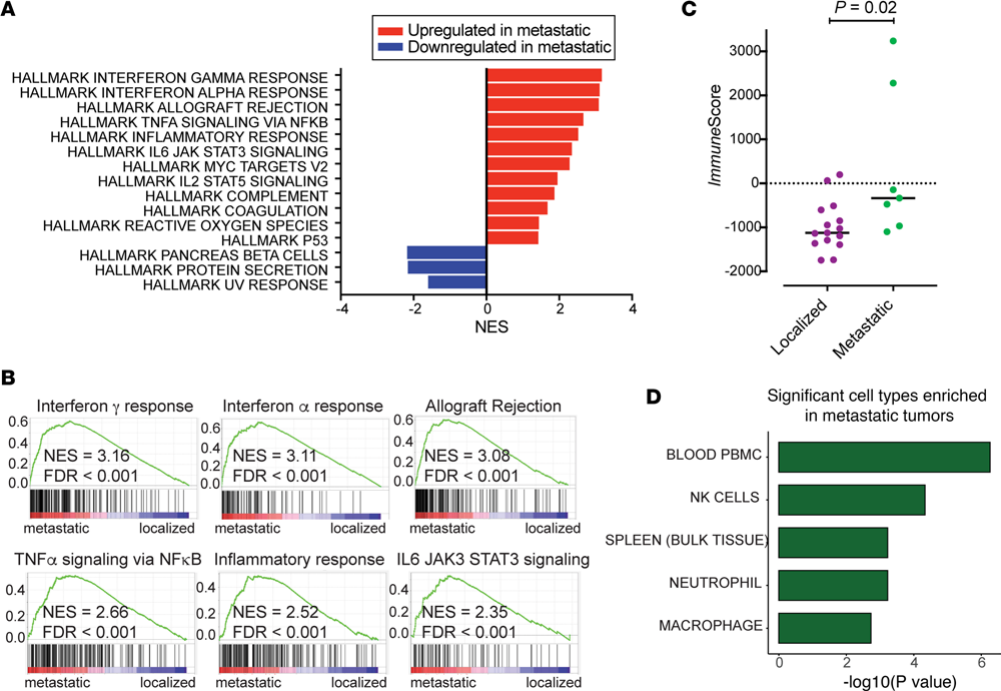
Interrogation of differentially expressed genes indicates activation of immune cell and inflammatory response pathways in metastatic PNETs. (**A**) GSEA pathway analysis showing the highest-ranked Hallmark pathways significantly altered (FDR *q* < 0.05) in metastatic versus localized PNETs. Upregulated (red) and downregulated (blue) gene sets in metastatic PNETs are shown with normalized enrichment scores (NES) on the *x* axis. (**B**) GSEA pathway analysis enrichment plots show significant enrichment of Hallmark gene sets related to immune system pathways. NES and FDR *q* are indicated. (**C**) ESTIMATE *Immune*Score analysis of patients in Cohort 1 comparing metastatic to localized PNETs. Horizontal bars indicate median. Compared with Wilcoxon rank-sum test. (**D**) Using the *EnrichR* enrichment analysis tool with the Human Gene Atlas library, immune cell types are shown to be significantly enriched in metastatic PNETs. The length of bar depicts comparative significance (by Wilcoxon rank-sum test).

Next, we compared biological pathways associated with differentially expressed genes using a gene set enrichment analysis (GSEA). Compared with localized tumors, metastatic PNETs exhibited significantly enriched scores in multiple signatures related to immune responses (Figure 3, A and B). Some of these hallmark pathways included IFN-γ response, IFN-α response, TNF-α signaling via NF-κB, inflammatory response, and IL-6/JAK/STAT3 signaling pathway that play crucial roles in immune cell signaling and inflammatory responses (31). In addition, other hallmark pathways that are linked with tumor regulation and response were upregulated in the metastatic tumors. Hallmark pathways in MYC targets and P53 suggested a link between cell growth control and metastatic tumors (32). Furthermore, enrichment for reactive oxygen species implicated an upregulation of oxidative stress responses among metastatic tumors. Downregulated pathways in metastatic PNETs included the hallmark pathways in pancreas β cells, protein secretion, and UV response. A higher fraction of insulinomas in localized PNETs in our cohort might have contributed to underrepresentation of pancreatic β cell–related pathways in metastatic tumors. Alternatively, this may represent a dedifferentiation process characterized by a loss of normal cellular processes among more aggressive tumors (33, 34).

Using estimation of stromal and immune cells in malignant tumor tissues using expression data (ESTIMATE) *Immune*Score and *EnrichR* analyses, we parsed out the contribution of specific stromal components to the upregulation of immune signaling signatures exhibited in metastatic tumors. The ESTIMATE *Immune*Score was significantly higher among metastatic compared with localized PNETs (*P* = 0.02; Figure 3C). There were 2 outliers in the metastatic tumors that were grade 2 tumors (Met_3 and Met_7), but they exhibited distinct clinical features to help explain the higher *Immune*Score compared with other specimens (Supplemental Table 1). In addition, *EnrichR* analysis determined an increase in gene signatures associated with peripheral blood mononuclear cells (PBMC) and NK cells among metastatic compared with localized tumors (Figure 3D). Together, these data suggest that immune cells may be more abundant in metastatic PNETs relative to localized tumors.

### Increased number of T cells infiltrating the primary site of metastatic PNETs.

**Figure 4:**
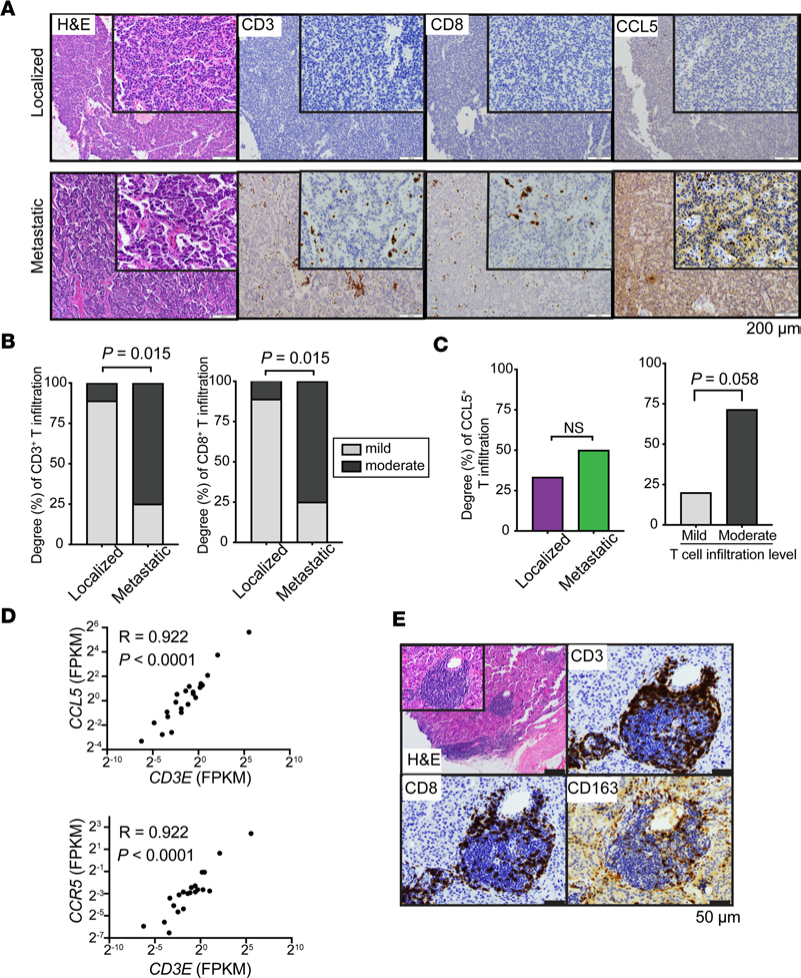
A more robust immune microenvironment identified in metastatic PNETs. (**A**) Representative images of H&E, CD3, CD8, and CCL5 IHC staining between metastatic and localized PNETs (*n* = 9 and 8 for each, respectively). Scale bar: 200 μm. (**B**) Degree (%) of CD3^+^ and CD8^+^ mild and moderate level of infiltration among localized and metastatic PNETs. Mild infiltration level is defined as < 5% among tumor cells, whereas moderate infiltration is defined as 5%–50% expression among tumor cells (35, 36). Metastatic PNETs had higher percentage of moderate infiltration of CD3^+^ and CD8^+^ T cells than localized tumors, which were associated more with a mild level of infiltration. *P* values were determined by Fisher’s exact test. (**C**) Degree (%) of CCL5^+^ T cell infiltration among localized and metastatic tumors (left) and among those with either mild or moderate T cell infiltration (right). Tumors with moderate T cell infiltration had higher CCL5 expression than those with mild infiltration, although statistical significance was marginally different (*P* = 0.058). (**D**) Scatterplots showing the range of associations (R) of *CCL5* versus *CD3* expression (top) and *CCR5* versus *CD3* expression (bottom). *P* values were determined by Spearman’s test. (**E**) Development of tertiary lymphoid structures stained for CD3, CD8, and CD163 was noted in the primary tumors of metastatic lesions (*n* = 2). Scale bar: 50 μm.

Given the elevated ESTIMATE *Immune*Score and PBMC/NK cell signatures identified in metastatic PNETs compared with localized tumors, we next sought to verify these findings via IHC analysis. Levels of CD3^+^ and CD8^+^ T cell infiltration in the primary sites of both the localized and metastatic PNET groups were analyzed by classifying infiltration levels into 3 groups (mild [<5%], moderate [5%–50%], and diffuse [>50%]) following guideline recommendations by evaluating the whole tumor area on a single section (Figure 4) (35, 36). This cohort (Cohort 2 in Figure 1A) included an additional 6 primary tumors that were not analyzed by RNA-Seq due to insufficient tissue available (Supplemental Table 2). We found that increased fractions of metastatic PNET tissues were associated with moderate levels of CD3^+^ and CD8^+^ T cells interspersed in the tumor, whereas the majority of localized PNET tissues displayed a mild degree of T cell infiltration (Figure 4, A and B). The difference between the degree of T cell infiltration between localized and metastasized PNET tissues was statistically significant (*P* = 0.015). None of the analyzed tumor tissues showed diffuse levels of T cell infiltration.

Chemokine and cytokine circuitries are key activators and traffickers of tumor-infiltrating T lymphocytes. Therefore, we investigated whether specific chemokines were correlated with T cell–infiltration levels. Among the known chemokines, *CCL5* (CC chemokine ligand 5, also known as RANTES), showed elevated mRNA levels in metastatic relative to localized tumors with marginal significance (log_2_ fold change [FC] = 2.69, adjusted *P* = 0.079) (Supplemental Figure 1). There was no significant difference in CCL5 expression between localized and metastatic tumors by IHC analysis (Figure 4C). However, we found that a subset of CD8^+^ cells exhibited a high level of CCL5 expression (Figure 4A), and the extent of T cell infiltration rather than metastatic status more closely correlated with the degree of CCL5 expression (Figure 4C). Accordingly, the RNA expression levels of *CD3* and *CCL5* showed a robust positive correlation (Figure 4D, top), supporting a close association between *CCL5* expression and T cell infiltration. Transcription level of *CCR5*, which encodes the receptor for CCL5, was also highly correlated with *CD3* expression levels (Figure 4D, bottom), illuminating that the CCL5/CCR5 axis plays a key role in T cell interaction within PNETs. CCR5 staining on PNET tissues yielded unusually high background (data not shown); thus, we were unable to assess the CCR5 protein expression status by IHC.

Tertiary lymphoid structures (TLS) are ectopic lymphoid aggregates that phenotypically resemble conventional secondary lymphoid organs and are commonly found at sites of chronic inflammation, including metastatic tumor sites (37). We discovered these dense aggregates of lymphocytes resembling TLS in 2 metastatic PNETs. Located primarily at the periphery of the tumor, these aggregates of CD3^+^ and CD8^+^ T cells and CD163^+^ macrophages provided further evidence that metastatic PNETs were associated with more immune activation than localized tumors, even at the primary tumor site (Figure 4E), suggesting tumor-specific differences in T cell recruitment.

### PNET-infiltrating T cells exhibit activated phenotype.

**Figure 5:**
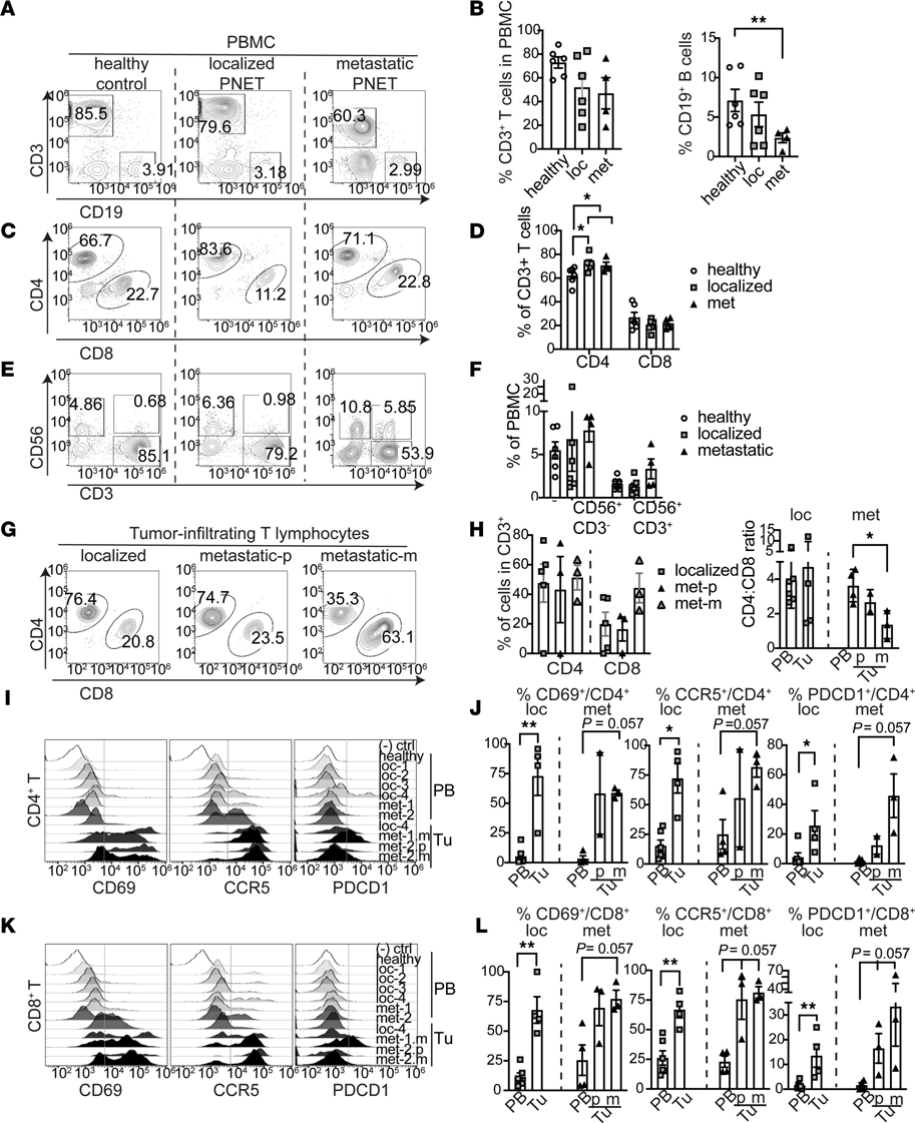
PNET-infiltrating T cells exhibit antigen-experienced phenotype. (**A**–**F**) PBMC analysis obtained from healthy volunteers and patients with localized and metastatic disease (*n* = 6 for healthy, *n* = 6 for localized, *n* = 4 for metastatic). (**A** and **B**) Representative flow cytometry and data plots of CD3^+^ T and CD19^+^ B cells in live PBMCs. (**C** and **D**) Flow cytometry plots and frequency analysis of CD4^+^ and CD8^+^ T cells gated on live CD3^+^ T cells. *P* value was determined by Mann-Whitney *U* test when comparing patients with all (localized and metastatic) disease to healthy controls. (**E** and **F**) Analysis of composition of peripheral CD56^+^ NK cells. (**G**) Flow cytometry plots of CD4^+^ and CD8^+^ T cells gated on live CD3^+^ T cells isolated from PNET tissues. (**H**) Summary data assessing percentages of gated CD4^+^ and CD8^+^ PNET-infiltrating T cells and analysis of CD4/CD8 ratio. Metastatic tumor tissues isolated from primary (p) and metastatic (m) sites were analyzed independently (*n* = 4 for localized, *n* = 3 for primary tumor of metastatic, *n* = 3 for metastatic site of metastatic PNET). (**I**) Representative flow cytometry plots of CD4^+^ T cells stained for CD69, CCR5, or PDCD1 expression. Gates were set by fluorescence minus 1 (MFO) control. Healthy donors and patients with localized (loc) or metastatic (met) tumors were indicated by numbers. T cells isolated from PBMCs (PB) and tumors (Tu) are marked on the right. Metastatic tumors isolated from metastatic (m) and primary (p) sites were analyzed separately. (**J**) Summary data of CD4^+^ T cell phenotypes. (**K**) Representative flow cytometry plots of CD8^+^ T cells analyzed for CD69, CCR5, or PD-1 expression. (**L**) Summary data of frequencies of T cell phenotypes. Kruskal-Wallis with Dunn’s multiple-comparison test was applied for 3 group comparisons (*P < 0.05; **P < 0.01). Data are shown as mean ± SEM.

Having demonstrated tumor-level differences in both gene expression and T cell infiltration between localized and metastatic PNETs, we sought to determine whether systemic inflammatory changes could also be identified in patients harboring PNETs. PBMCs from both healthy donors and patients with PNETs were isolated and analyzed to determine the immune cell profile by flow cytometry. We found that the frequencies of circulating CD3^+^ T cells did not differ across healthy donors or those with either localized or metastatic disease, but the percentage of composition of peripheral CD19^+^ B cells was lower among patients with metastatic disease compared with healthy controls (Figure 5, A and B). The percentages of CD4^+^ T cells were greater in patients with both localized and metastatic disease than in healthy controls, but this difference was not observed in CD8^+^ T cells (Figure 5, C and D). Levels of peripheral CD56^+^ NK cells, regulators of the innate immune system with potent cytolytic antitumorigenic activity (38), did not differ across cohorts or by CD3^+^ coexpression (Figure 5, E and F).

Next, we determined the characteristics of tumor-infiltrating T cells that we isolated from surgical specimens from both metastatic and localized PNETs. The majority of PNET tissues were infiltrated with CD3^+^ T cells that were detectable by flow cytometry analysis. The gating scheme is described in Supplemental Figure 2. Additionally, we found that PNET metastatic sites were significantly enriched with cytotoxic CD8^+^ T cells, and the CD4/CD8 ratio of T cells isolated in metastatic sites were skewed from its ratio observed in peripheral blood, indicative of a tumor-driven immune response (Figure 5, G and H).

To discern whether alterations of T cell compartments were associated with T cell activities in the TME, we evaluated the phenotypes of tumor-infiltrating T cells and compared them with those of peripheral T cells (Figure 5, I–L). Specifically, expression levels of CD69, CCR5, and PDCD1 were assessed. CD69 has been identified as a marker of lymphocyte activation and tissue retention; CCR5 has been shown to be an important marker for both mediation of cognate cytotoxic T cell recruitment to tumors and for the maximal priming and generation of cytotoxic T cells during antitumor responses; and PD-1 has been associated with chronic antigenic activation leading to T cell exhaustion (39–42). The frequencies of activated T cells in the PBMC of healthy controls and patients with PNETs were in similar ranges (Supplemental Figure 3). However, tumor-infiltrating CD4^+^ and CD8^+^ T cells showed much higher percentages of CD69, CCR5, and PDCD1 expression when compared with circulating T cells (Figure 5, I and K). This difference was significant in localized tumors (*P* < 0.01), and a similar trend was observed among metastatic PNETs with marginal significance (Figure 5, J and L). Overall, our findings show that, despite indistinguishable differences in the activity of peripheral T cells between healthy controls and those with PNETs, T cells within PNET TME display higher levels of activation, trafficking, and exhaustion than in the periphery.

### Histone deacetylase inhibitor increased tumoral CCR5 expression, leading to T cell migration.

**Figure 6:**
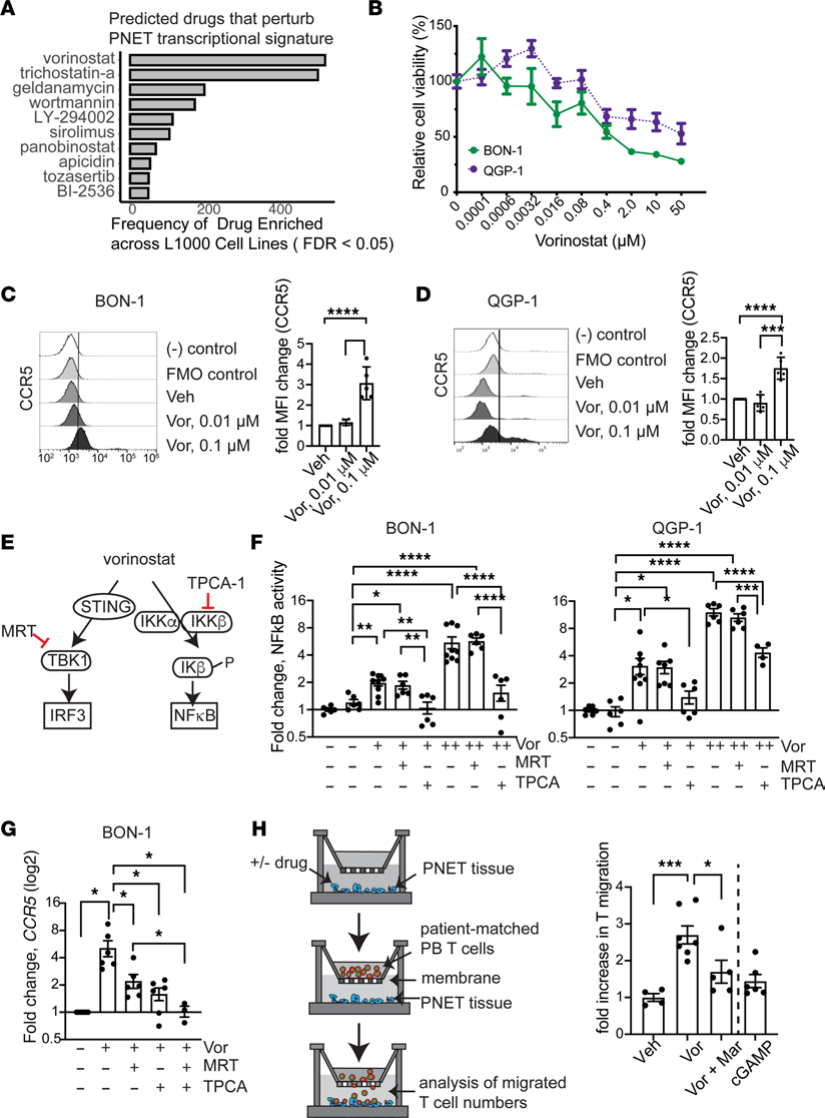
Vorinostat regulates tumoral CCR5 expression via the NF-κB pathway, which augments T cell recruitment to PNET. (**A**) Graphical representation of L1000 drug screen. (**B**) Cell growth assay of PNET cell lines upon exposure to vorinostat (*n* = 9 for each cell line from 3 independent experiments). (**C** and **D**) Representative flow cytometry plots of CCR5 expression. PNET cell lines were treated with subtoxic doses of vorinostat (vor). Fold increase of mean fluorescence intensity (MFI) was obtained by normalization of CCR5 expression in vor-treated cells by vehicle-treated (veh-treated) cells. Experiments were repeated at least 2 times independently (*n* = 4–5). (**E**) Model for vorinostat modulation of canonical STING and NF-κB pathways. Inhibitors MRT67307 (MRT) and TPCA-1 block STING and NF-κB pathways, respectively. (**F**) Measurement of NF-κB activity. PNET cell lines were treated with vor alone (+ and ++ indicates 0.1 μM and 0.5 μM, respectively) or in combination with MRT (200 nM) or TPCA-1 (20 μM). Luminescence units were normalized to veh-treated control group (*n* = 6–9 from 3 independent experiments). (**G**) RT-PCR analysis of *CCR5* expression in BON-1 cells following 24-hour treatment with vor (0.1 μM), MRT (200 nM), and TPCA-1 (20 μM). Fold change was normalized to veh-treatment condition (*n* = 6 from 2 independent experiments). (**H**) Left: T cell migration assay was performed with fresh metastatic PNET tissue and patient-matched peripheral T cells. Right: Relative number of migrated T cells normalized to veh-treatment group. In treatment groups, PNET tissues were exposed to vorinostat (0.05 μM) for 72 hours prior to autologous T cell addition. Competitive CCR5 antagonist maraviroc (10 μM) or cGAMP (1 μM) was added, as indicated. Data were combined from 2 patients with metastatic PNETs (*n* = 4–7). Statistical analysis was performed using 1-way ANOVA with Tukey’s multiple-comparison test (*P < 0.05; **P < 0.01; ***P < 0.001; ****P < 0.0001). Data are shown as mean ± SEM.

Given the differential expression identified between localized and metastatic PNETs, we analyzed RNA-Seq data with an L1000 drug-induced signature profile to identify pharmacologic agents that reverse the PNET metastatic transcriptional signature, thus potentially targeting metastatic disease (43). Vorinostat and trichostatin A, both inhibitors of the histone deacetylase (HDAC) class of enzymes (44), were the most frequently recurring drugs predicted to perturb the metastatic PNET transcriptional signature (Figure 6A).

Therefore, we investigated the cellular and molecular changes induced by vorinostat treatment on 2 PNET cell lines — BON-1 and QGP-1 — and these cell lines have previously been used as models for metastatic and localized PNETs, respectively (45). Both PNET cell lines were sensitive to increasing concentrations of vorinostat, with BON-1 cells appearing slightly more sensitive than QGP-1 (Figure 6B).

In addition to cellular toxicity elicited by vorinostat, we interrogated whether vorinostat induces changes in chemokine expression that may modulate TME. Given the role of CCR5 in mediating T cell recruitment and our findings of higher CCL5 expression levels noted in PNETs with a higher infiltration level (Figure 4, A and C), we explored the effects of vorinostat on CCR5 expression in PNET cell lines. Following treatment with sublethal dose of vorinostat, both BON-1 and QGP-1 cell lines showed significant increases in the expression of CCR5, as measured by flow cytometry (Figure 6, C and D). In addition, CCL5, the ligand to CCR5, displayed elevated expression in both cell lines, although the CCL5 fold induction level was lower than CCR5 in BON-1 (Supplemental Figure 4).

Although chemokine expression in PNETs has been poorly studied, upstream signaling pathways including NF-κB and canonical cyclic GMP-AMP synthase/stimulator of interferon genes (STING) pathways have been shown to regulate CCR5 and CCL5 expression in other types of cancer (46). Correspondingly, previous reports implicate vorinostat for activating these signaling pathways (47) (Figure 6E). Therefore, we investigated the specific signaling pathway activated by vorinostat to induce CCR5 expression. In both BON-1 and QGP-1 cell lines, vorinostat treatment augmented NF-κB activity in a dose-dependent fashion (Figure 6F). Cotreatment of vorinostat with TPCA-1 (inhibitor of IKKβ that activates canonical NF-κB pathway) reduced NF-κB activity significantly (Figure 6F); however, MRT67307 (inhibitor of TBK1, which is a component of canonical STING pathway) cotreatment did not reduce vorinostat-induced NF-κB activity, illuminating the dominant effect of vorinostat on modulating NF-κB activity in PNET cells. Vorinostat-induced *CCR5* mRNA expression was also significantly diminished by the TPCA-1 or MRT67307 cotreatment (Figure 6G). Combination treatment with both TPCA-1 and MRT67307 inhibitors reduced vorinostat-induced *CCR5* expression almost to its basal level, with a significant reduction compared with MRT67307 monotreatment. These data show that both canonical NF-κB and STING pathways mediate vorinostat-induced *CCR5* expression, but with a stronger contribution by NF-κB pathway.

Next, we examined whether vorinostat treatment impacts T cell migration in a more biologically relevant setting. We mechanically dissected freshly resected metastatic PNET tissue into approximately 1–2 mm^3^ pieces, added vorinostat as pretreatment, and assessed autologous peripheral T cell migration across a semipermeable membrane toward the tumor (Figure 6H). Vorinostat treatment increased T cell migration substantially; this migration was mitigated by cotreatment with maraviroc, a competitive CCR5 antagonist, demonstrating that vorinostat-induced T cell migration was dependent on CCR5. Treatment with a vorinostat of the same dose examined on PNET cell lines did not induce CCR5 expression substantially in peripheral T cells, implying that PNET tissues are more responsive to vorinostat than T cells (Supplemental Figure 5). In addition, T cell migration was not stimulated by an addition of 2’,3’-cyclic guanosine monophosphate-adenosine monophosphate (cGAMP), a direct agonist of the canonical STING pathway mediated by IFN regulatory factor 3 (IRF3) (Figure 6H). Taken together, our data suggest that vorinostat modulates PNET immunogenicity by producing chemokine CCR5, which augments T cell recruitment into the TME.

### Increased T cell infiltration to mouse PNET after treatment with HDAC inhibitor.

**Figure 7:**
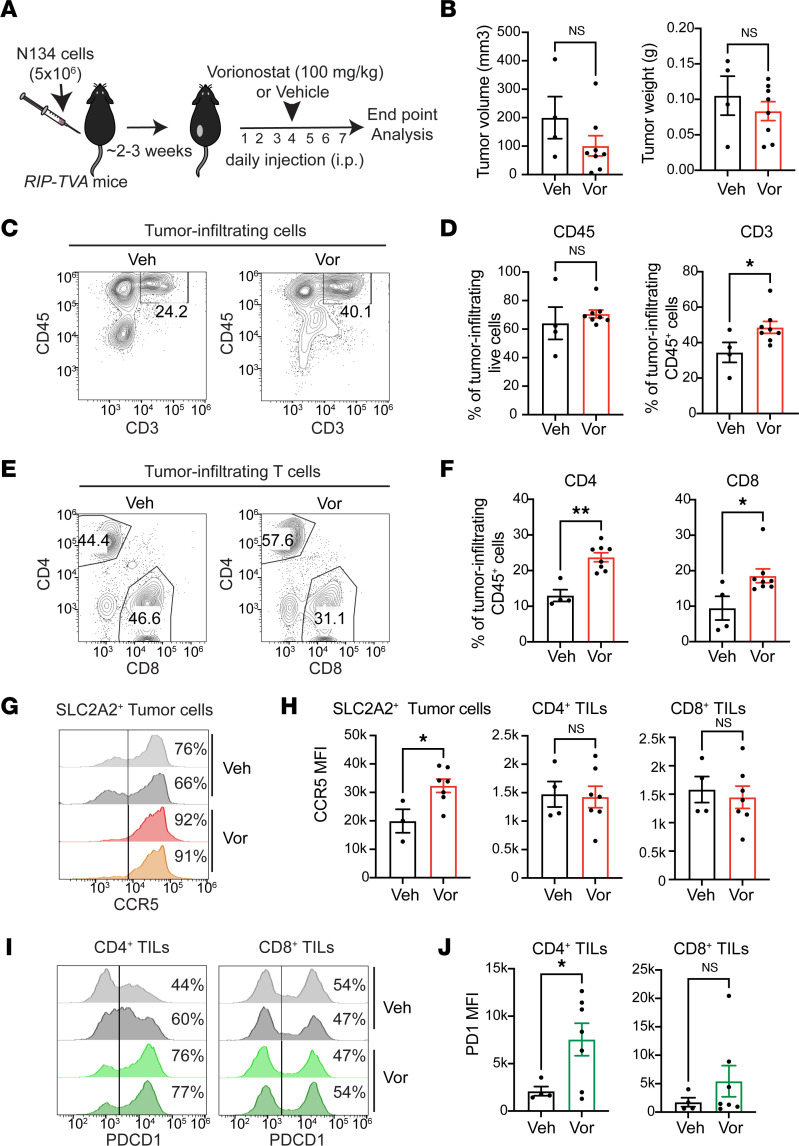
Vorinostat increases T cell recruitment and activation to the tumor in a mouse model of PNET. (**A**) Schematic view of mouse PNET establishment and treatment with vorinostat or vehicle control. (**B**) End-point analysis of tumor volume and weight (*n* = 4 and 8 for vehicle [veh] and vorinostat [vor] treatment groups, respectively). (**C**) Representative flow cytometry plots analyzing CD45 and CD3 expression. Events were gated on live cells only. Percentages of gated cells are shown on the plots. (**D**) Percentages of CD45^+^ hematopoietic and CD3^+^ T cells isolated from mouse PNET tissues are analyzed. (**E**) Representative flow cytometry plots show CD4 and CD8 expression on live tumor-infiltrating CD45^+^CD3^+^ T cells. (**F**) Summary plots of frequencies of CD4^+^ or CD8^+^ cells among CD45^+^ hematopoietic cells in the tumor. (**G**) Representative flow cytometry histogram plots displaying CCR5 expression on SLC2A2^+^ live tumor cells. (**H**) Left: Summary plots showing MFI of CCR5 expression on PNETs isolated from mice either treated with veh or vor (*n* = 3 and 7 for veh- and vor-treatment groups, respectively; 1 veh-treated PNET sample with < 2% SLC2A2 expression was excluded from the analysis). Middle and right: Summary plots of CCR5 MFI on tumor-infiltrating CD4^+^ and CD8^+^ T cells (*n* = 4 and 7 for veh- and vor-treatment groups, respectively). (**I**) Representative flow cytometry plots of PDCD1 expression on CD4^+^ or CD8^+^ PNET-infiltrating T cells. (**J**) Bar plots show PDCD1 MFI of CD4^+^ or CD8^+^ PNET-infiltrating T cells (*n* = 4 and 7 for veh- and vor-treatment groups, respectively). (**B**, **D**, and **F**) Statistical analysis was performed by Mann-Whitney *U* test. (**H** and **J**) Statistical analysis was performed by Student’s *t* test (*P < 0.05; **P < 0.01). Data are shown as mean ± SEM.

Finally, we examined whether the TME modulation by vorinostat can impact T cell recruitment in vivo. We have recently developed a syngeneic mouse model of PNET immunocompetent mice (48). Here, we s.c. implanted a mouse PNET cell line, N134 (derived from a mouse model, *RIP-Tag; RIP-tva*; ref.^49^) into the flanks of syngeneic *RIP-tva* mice (C57BL/6 background) (Figure 7A). The PNETs in this mouse model are induced by the expression of SV40 T antigen (Tag) oncogene in islet β cells. This mouse model has served as a valuable model to represent human PNET development with similar molecular profiles (33) and sensitivity to targeted molecules, such as VEGF and MTOR (2). After tumors reached ~100 mm^3^, mice were administered vorinostat or vehicle control daily for 1 week and then analyzed before animals died of hypoglycemia (Figure 7A). We found a trend that vorinostat reduced tumor size and weight, although it did not reach statistical significance (Figure 7B). We observed that vorinostat treatment led to significantly greater CD3^+^ T cell infiltration, but not infiltration of all CD45^+^ hematopoietic cells, within the TME compared with the vehicle control (Figure 7, C and D). The frequencies of both CD4^+^ T and CD8^+^ T cells were statistically greater in the vorinostat-treated mice (Figure 7, E and F).

We examined whether the expression of CCR5 is increased in mouse PNET upon vorinostat administration, like the observations made in human PNET tissues. The expression of SLC2A2 (also known as glucose transporter 2 [GLUT2]) was used to identify insulinoma cells (33). The frequency of SLC2A2 between 2 cohorts were not significantly different (Supplemental Figure 6). The CCR5 expression level was markedly increased in SLC2A2^+^ cells when mice were treated with vorinostat (Figure 7, G and H). This level of elevation was not observed in tumor-infiltrating T cells (Figure 7H), supporting prior findings in human PNET tissues that PNET cells are more sensitized to vorinostat than T cells. Meanwhile, PNET-infiltrating CD4^+^ T cells, but not CD8^+^ T cells, showed enhanced expression of PDCD1 (Figure 7, I and J). Our in vivo findings demonstrate that vorinostat increases CCR5 expression in PNETs significantly, which may contribute to T cell recruitment and activation in the tumor.

## Discussion

Understanding the tumor-immune microenvironment is critical to enhancing immunotherapies for PNETs. The data presented here demonstrate that the tumor-immune microenvironment is different between localized and metastatic PNETs in the primary pancreas tissue. We identify significantly activated immune cell signaling pathways in metastatic compared with localized PNETs by RNA-Seq analysis. Correspondingly, more metastatic PNETs are associated with moderate levels of T cell infiltration than localized PNETs. Tumor-infiltrating T cells isolated from PNET sites — both localized and metastatic — show higher levels of antigen-dependent activation than peripheral T cells. In silico analysis identifies the HDAC inhibitor vorinostat as the drug most likely to perturb metastatic PNET transcriptional signatures. We then demonstrate that vorinostat not only inhibits PNET cell growth, but also modulates PNET cell expression of CCR5 primarily via the NF-κB pathway. This enhanced chemokine expression results in increased T cell migration toward patient-derived PNET tissue and was supported by findings in a PNET mouse model, suggesting that modulation of the TME among PNETs may serve a clinical benefit in future treatment algorithms.

In other solid tumors, the immunogenic TME has been shown to greatly contribute to the clinical response following checkpoint blockade therapy and/or therapeutic cancer vaccines and to, thus, affect prognosis (50, 51). Among PNETs, more advanced stages appear to have a significantly higher degree of CD3^+^ PDCD1^+^ T cell infiltration (25, 29); however, there have been contradictory reports regarding the degree of TIL infiltration within PNETs and prognosis. In a series of studies, high TIL infiltration has been associated with higher-grade tumors and poorer outcomes among patients with neuroendocrine neoplasms (29, 52), whereas higher density of TILs has been associated with longer disease-free survival in other studies (25, 30). Recent retrospective analyses of over 100 PNET cases have illuminated that well-differentiated PNETs represent immune-responsive microenvironments. High intratumoral CD8^+^ infiltration (30), HLA class I expression (30), and presence of TLS (53), independently, were associated with better prognosis. PNETs have also been noted to exhibit a high level of tumor-associated macrophage infiltration, which may contribute to creating an immunosuppressive TME by releasing cytokines that promote the expression of inhibitor checkpoint proteins in T cells (30, 54, 55). Our findings support the growing body of literature that suggests a more complex TME among PNETs, with definitive increases in the percentage of CD69^+^, CCR5^+^, and PDCD1^+^ T cells among localized, and likely metastatic, tumors compared with peripheral blood. Our expression marker analysis identifies these PNET-infiltrating T cells as antigen experienced, activated, and, ultimately, exhausted. Our analysis also demonstrates a potential avenue of immunogenic optimization via TME modulation with vorinostat.

Immune checkpoint inhibitors such as pembrolizumab are thought to minimize tumor cell escape from immune surveillance by elevating antitumor T cell immunity (56). Unfortunately, despite the increased PD-L1 expression observed across NET sites that has been correlated with tumor grade (29, 57), results from the KEYNOTE-028 trial, in which patients with PD-L1 and well-differentiated PNETs were treated with pembrolizumab, were tepid, similar to the results of a trial with spartalizumab (26, 27). The reasons for limited immune checkpoint therapy efficacy are varied but may be partly due to restricted actionable T cell numbers.

Recently, alternative strategies to elicit T cell responses by inducing changes in tumor-associated immune cell activities have shown significant effects when combined with immune checkpoint therapy. Activation of innate immune signaling through the cGAS/STING pathway has been proposed as an attractive approach in enhancing the responsiveness of existing immunotherapies (58, 59). Our study demonstrates that vorinostat, a potent class I/II HDAC inhibitor, may represent another such potential therapeutic agent. Beyond its marked effects on PNET cell viability, as well as its generalized effects on reversing the metastatic PNET signature, vorinostat was found to modulate tumoral CCR5 expression in vitro, a regulation that serves as the nexus of T cell migration toward metastatic PNET tissue. While CCR5 is naturally expressed on T cells, its overexpression on cancer cells themselves can also be a consequence of oncogenic transformation and has been found in many solid organ tumors, including those of breast, colorectal, esophageal, gastric, and pancreatic origin (60, 61). Herein, we have shown that vorinostat treatment of PNET cell lines resulted in increased CCR5 expression. Within our study, peripheral T cells were found to have low CCR5 expression in contrast to T cells isolated from PNETs. Because cotreatment of the PNET tissues with vorinostat and maraviroc mitigated peripheral T cell recruitment toward the PNET tissues that was induced by vorinostat alone, our findings implicate that vorinostat enhances T cell recruitment via engagement of CCR5, which is most likely expressed on the tumor cells. These alterations may increase CCL5^+^ T cell infiltration into the CCR5^+^ PNETs. Administration of vorinostat also resulted in an increased proportion of T cells in the TME of a PNET mouse model, demonstrating a conserved mechanism for the effect of vorinostat on PNETs. Future studies determining the underlying interactions between tumor and immune cells will help our understanding of how the CCR5/CCL5 axis plays a major role in T cell infiltration into PNETs.

Targeting the HDAC pathway in PNETs has previously been proposed for its ability to induce toxicity (45, 62, 63). HDAC5 overexpression in PNET tissue has been associated with poor clinical outcomes, including low overall and disease-free survival rates (62, 64). Monotherapy of pan-HDAC inhibition in a phase II clinical trial has reported durable stable disease in 4 of 5 patients with PNETs (65). Combination therapy with vorinostat and pembrolizumab is currently being explored in clinical trials for advanced/metastatic non–small cell lung cancer and metastatic head and neck squamous cell carcinomas with a positive preliminary outcome (59, 66), lending a possible therapeutic avenue for the treatment of patients with metastatic PNETs. Our in vivo findings in a mouse model of PNET also substantiate that combination therapy of PD-1 inhibitor and vorinostat may be more effective in controlling tumor growth than either monotherapy.

Emerging studies show that immunopeptidomes, which elicit antitumor responses, can be derived from multiple sources including aberrant noncoding RNA transcripts, alterations in RNA splicing and translation, and posttranslational changes, as well as canonical nonsynonymous mutations (67). A recent study in acute myeloid leukemia has demonstrated a high correlation between the occurrence of tumor-specific epitopes and mutations in epigenetic modifiers (68). Although PNETs are associated with low mutational burden, epigenetic aberrations commonly found in PNETs (69) may indeed drive the activity of targetable antitumor T cells. Identification of PNET immunogenic antigens and isolation of tumor-specific T cells, accomplished by selecting for activity markers and/or PDCD1 (70), may augment synergy with other cancer immunotherapy approaches, including adoptive T cell therapies.

In summary, our integrative study on rare PNET tumors demonstrates that metastatic PNETs provide a permissive environment for activating tumor-infiltrating T cells — a finding that has not been appreciated previously. Moreover, metastatic PNET tissues are responsive to an HDAC inhibitor, with subsequent T cell chemoattraction toward tumor tissue. Mechanistically the chemokine receptor CCR5 mediates PNET and T cell interactions, and HDAC inhibition works through the NF-κB pathway to regulate CCR5 expression. A comprehensive understanding of the TME and its regulatory network may further guide specific therapeutic regimens to achieve the goal of prolonging survival in patients with PNETs, including those not amenable to surgical resection.

## Methods

### RNA-Seq.

Total RNA was extracted from flash-frozen tumor tissues using the materials and protocol provided in the RNeasy mini kit (catalog 74106, Qiagen). Total RNA was used for TruSeq stranded library generation, and paired end clustering with 51 base pairs × 2 ends of sequencing was performed on HiSeq 2500/4000 (Illumina) by the Genomics Core Facility of the Weill Cornell Medicine Core Laboratories Center. Raw and processed RNA-Seq data are openly available via Gene Expression Omnibus (GEO; accession no. GSE178398).

### Bioinformatics.

Raw sequenced reads were aligned to the human reference genome (version hg19 from UCSC) using STAR (version 2.4.2) aligner. Aligned reads were quantified against the reference annotation (hg19 from UCSC) to obtain fragments per kilobase per million (FPKM) and raw counts using *CuffLinks* (v 2.2.1) and *HTSeq*, respectively.

Differential expression to compare expression profiles of respective groups was performed on normalized raw counts using the *limma* package in R. Genes with FDR-adjusted *P* < 0.05 were significantly differentially expressed. Pathway analysis using GSEA software from Broad Institute was used to identify functions of differentially expressed genes (71). Genes were ranked by the t-statistic value obtained from comparisons, and the preranked version of the tool was used to identify significantly enriched biological pathways. Hierarchical data clustering and PCA were performed to classify the samples based on gene expression profiles with unsupervised clustering. Both methods were performed on the log_2_ transformed FPKM expression values in R statistical software. Pathways enriched with FDR *q* < 0.05 were significant. Additionally, gene name–based pathway analysis was carried out using the online webtool ConcensusPathDB (cpdb) and ENRICHR (72, 73). The LINCS L1000 database was used to infer transcriptional expression-based drug reversal (43). We used a GSEA approach to test for enrichment of a drug-modulated transcriptional signature in a list of genes ranked by log_2_ FC from metastatic versus localized comparison. Drugs were ranked by recurrence of hits to identify and prioritize downstream candidates. We used the ESTIMATE tool to compute the *Immune*Score and stromal score based on the gene expression profiles (74). Comparison of the ESTIMATE score for *Immune*Score was made using Wilcoxon test.

### Cell lines.

Two human PNET cell lines, QGP-1 and BON-1, were grown at 37°C under 5% CO_2_ (75). QGP-1 was established from a primary somatostatin producing PNET (76), and BON-1 was established from a lymph node metastasis of a PNET (77). Both cell lines were grown in RPMI-1640 media (catalog 11875093, Thermo Fisher Scientific) supplemented with 10% FBS. Cell lines were confirmed negative for *Mycoplasma* prior to the study with the kit (catalog LT07-118, Lonza) following the protocol provided by the manufacturer. Cell lines were maintained from cryopreserved stocks made at low passage numbers, and all cells used in experiments were of a passage number < 20.

### Drug sensitivity assay.

Vorinostat (SAHA, MK0683) (catalog S1947, Selleck Chemicals) was dissolved in dimethyl sulfoxide (DMSO), and stock solutions were stored at –80°C. QGP-1 and BON-1 cells were plated in at a density of 10,000 cells per well, within 200 μL of phenol-free RPMI media per well in 96-well flat-bottomed culture dishes. After incubation overnight at 37°C, media were replaced with media containing vorinostat at the indicated concentrations and incubated for 72 hours. Each condition was performed in triplicate. Cell viability was assessed using the Vybrant MTT Cell Proliferation Assay Kit (catalog V13154, Thermo Fisher Scientific). Measurement of absorbance using a microplate reader was performed at 540 nm.

### IHC.

Pancreatic tumor samples were selected from patients who underwent surgical resection of either localized (*n* = 9) or metastatic (*n* = 9) well-differentiated PNETs. Twelve patients from Cohort 1 were included in this analysis, along with all patients from Cohort 2 (Figure 1 and Supplemental Table 2). Formalin-fixed, paraffin-embedded human tissue blocks were cut at 5 μm–thick intervals, and consecutive slides were stained for H&E, CD3 (catalog PA0553, clone LN10, Leica), CD8 (catalog PA0183/clone 4B11, Leica), CCL5 (catalog ab9679, Abcam) at the Translational Research Lab at WCM. Board-certified endocrine pathologists (TS, DK) reviewed each case to ensure correct diagnosis and to determine the tumor extent, CD3, and CD8 staining.

### Peripheral blood and tumor tissue processing.

Whole blood samples were obtained by venipuncture and collected in EDTA-containing vacutainer tubes (catalog 22-253-145, BD Biosciences). Whole blood samples were also obtained from 6 healthy control subjects. PBMCs were isolated over Ficoll-Paque PLUS (catalog 17144002, GE Healthcare) and cultured in TexMACS GMP medium (catalog 130-097-196, Miltenyi Biotec) supplemented with 5% human AB serum (catalog H4522, Sigma-Aldrich) at a density of 1 × 10^6^ to 2 × 10^6^ cells/mL. Nonadherent PBMCs were recovered after overnight culture and washed twice with PBS before flow cytometry analysis. Surgically resected fresh tumor tissues were finely minced and were seeded in a 6-well plate in RPMI-1640 media supplemented with 10% FBS and 100 U/mL penicillin/streptomycin (Pen/Strep). After overnight culture, single-cell suspensions were prepared after short trypsin treatment and filtered using 70 μm cell strainer (catalog 352350, Falcon). Tumor-infiltrating T cells and tumor cells were further washed twice prior to flow cytometry analysis.

### T cell migration assay.

A T cell migration assay was performed utilizing Corning Matrigel Invasion Chamber (catalog 354481, Corning Inc.) according to the manufacturer’s protocol. Briefly, surgically resected fresh tumor tissues were finely minced and were seeded in a 24-well plate in RPMI-1640 media supplemented with 10% FBS and 100 U/mL Pen/Strep. Certain wells had chemical treatment with vorinostat (0.05 μM) alone or in combination with 10 μM of maraviroc (catalog S2003, Selleck Chemicals, dissolved in DMSO) or cGAMP sodium salt (catalog SML1229, Sigma-Aldrich) for 72 hours. Meanwhile, patient-derived T cells, isolated as above, were activated using CD3/CD28 Dynabeads (catalog 1161D, Thermo Fisher Scientific) for 2 days. After 72-hour treatment, invasion chambers were rehydrated with 1 mL of RPMI-1640 media supplemented with 10% FBS for 2 hours. Activated T cells were stained with Calcein AM dye (catalog C1430, Thermo Fisher Scientific) for 1 hour, 100,000 T cells suspended in 500 μL of TexMACS GMP medium were applied to the top of the transwell, and maraviroc 10 μM was redosed. After 12 hours, 0.24 × 10^5^ CountBright Absolute Counting Beads (catalog 36950, Thermo Fisher Scientific) were applied to bottom of the transwell, and all contents in the bottom well were prepared for flow cytometry. Live green-fluorescent T cells and beads were gated based on scatter and fluorescence emission. The number of cell events was determined based on the recovered bead counts in each well, and each experimental group was normalized to the control T cell group, of which a tumor sample was pretreated with DMSO.

### Flow cytometry analysis.

Staining of human PBMCs and tumor-infiltrating T cells was performed on ice for 20 minutes in 1× HBSS containing 2% FBS and 0.1% BSA. Anti–human CD3-PECy5/CD4-PE/CD8-FITC cocktail (catalog 319001, clones UCHT1, RPA-T4, RPA-T8), Alexa Fluor 647 anti–human CD69 (catalog 310918, clone FN50), APC-conjugated CCR5 (catalog 359122, clone J418F1), and APC/Fire 750–conjugated PDCD1 (catalog 329953, clone EH12.2H7) antibodies were from BioLegend. Live cell gating was determined by Calcein Blue AM (catalog C1429, Thermo Fisher Scientific) uptake, from which subpopulations of cells were selected (Supplemental Figure 2).

Staining of mouse PNET-infiltrating T cells was performed on ice for 20 minutes in 1× PBS containing 2% FBS. After blocking with TruStain FcX PLUS (catalog 156604), cells were stained with antibodies containing PE-Cyananine7 anti–mouse CD45 (catalog 103113, clone 30-F11), APC anti–mouse CD3 (catalog 100235, clone 17A2), PE anti–mouse CD4 (catalog 100407, clone GK1.5), FITC anti–mouse CD8 (catalog 100705, clone 53-6.7), PE–Cyananine 7 anti–mouse CCR5 (catalog 107018, clone HM-CCR5), APC–Cyananine 7 anti–mouse PDCD1 (catalog 135224, 29F.1A12), and APC anti–mouse SLC2A2 (catalog FAB1440A, clone 205115). APC anti–mouse SLC2A2 was from R&D systems; all other antibodies were from BioLegend. Live cell gating was determined by Propidium Iodine (catalog P4864, Sigma-Aldrich) negative uptake. Gates for positively stained cells were set using fluorescence minus 1 (FMO) control. Flow cytometry data were acquired using Gallios flow cytometry (Beckman Coulter) and analyzing using FlowJo (BD Biosciences) software.

### NF-κB luminescence assay.

An NF-κB Cignal Reporter Assay (Qiagen) was performed according to the manufacture’s protocol. Briefly, 0.1 × 10^6^ BON-1 and QGP-1 cells were transfected with 50 μL of NF-κB firefly luciferase reporter lentivirus (catalog CLS-013L, Qiagen) and allowed to expand. Transfected cells were selected by treatment with puromycin (2.0 μg/mL) for 2 weeks, redosed every 48–72 hours. Cells were treated with vorinostat with or without MRT67307 (TBK1/IKKε inhibitor) (catalog 50-630-60001, Sigma-Aldrich) and TPCA-1 (IKKβ inhibitor) (catalog T1452, Sigma-Aldrich) for 24 hours for QGP-1 and 48 hours for BON-1 cells in RPMI-1640 media supplemented with 10% FBS and 150 μg/mL of D-luciferin (Gold Biotechnology). Luminescence signal was measured using a TECAN Infinite M1000 PRO microplate reader (Tecan Group Ltd).

### Reverse transcription PCR (RT-PCR).

RNA was extracted from BON-1 and QGP-1 cell lines using RNeasy Mini Kit according to the manufacture’s protocol. First-strand cDNA synthesis was performed using 1 μg of each RNA sample primed with SuperScript reverse transcriptase III (catalog 18080092, Thermo Fisher Scientific) with random hexamers (catalog N8080127, Thermo Fisher Scientific). A reaction mixture containing 2 μL of cDNA template, 5 μL TaqMan Universal PCR master mix (catalog 4444557, Applied Biosystems), 3.5 μL nuclease-free water, and 0.5 μL primer probe mixture was amplified following the manufacture’s protocol. *CCR5* gene expression (probe Hs99999149_s1, Thermo Fisher Scientific) was measured in triplicate and normalized relative to the housekeeping gene β*-*actin (Hs99999903_m1, Thermo Fisher Scientific). Reference normalized expression measurements (ΔCt) in triplicate were used for statistical analysis. Gene expression values were calculated according to the ΔΔCT method.

### Mouse studies.

N134 cells (5 × 10^6^ cells mixed with DMEM with 2% FBS in a total volume of 100 μL) were s.c. injected into *RIP-TVA* (C57BL/6 background) mice on the bilateral flanks (48). Tumor formation was monitored, and tumor volume (mm^3^) was based on caliper measurements and calculated by the modified ellipsoidal formula (W^2^ × L)/2 × 1,000, where L is the long diameter and W is the short diameter. When tumor sizes reached 100 mm^3^ on average, mice were treated with vorinostat 100 mg/kg (formulated in DMSO/Kolliphor EL [C5135, Sigma-Aldrich]/Saline solution with the ratio of 1:1:8) or vehicle control (DMSO/Kolliphor EL/Saline solution with a ratio of 1:1:8) by i.p. injection daily for 7 days. The mice were euthanized 1 day after the final treatment, and the tumors were harvested in PBS and weighed after PBS removal. Subsequently, the tumors were finely minced and digested with 300 U/mL of collagenase type III (catalog 26970S, Cell Signaling Technology) for 1 hour in a 37°C incubating shaker and then strained through a 70 μm filter to achieve a single-cell suspension. The cells were then pelleted, and RBCs were removed by treatment with ACK Lysing Buffer (A10492-01, Thermo Fisher Scientific) for 5 minutes on ice. The resultant single-cell suspension was then prepared for flow cytometry as above.

### Statistics.

Patient clinical factors were compared between the localized and metastatic PNET cohorts selected for RNA-Seq analysis. Progression-free survival curve was analyzed by Gehan-Breslow-Wilcoxon test. Descriptive statistics were performed using 2-tailed Student’s *t* test and Fisher’s exact test for continuous parametric and categorical variables, respectively. Wilcoxon rank-sum test was used to compare the ESTIMATE *Immune*Score between localized and metastatic cohorts. IHC expression levels between the localized and metastatic samples were compared with Fisher’s exact test. Correlation analysis for mRNA expression was determined by Spearman’s test. Frequencies of specific cell populations and tumor size in each group (Figures 5 and 7) were compared by Mann-Whitney *U* test for 2-group comparisons and Kruskal-Wallis with Dunn’s multiple-comparison test when more than 2 groups were compared. Experiments comparing fold increase of molecule expression, gene activity, and T cell migration were analyzed using 1-way ANOVA with Tukey’s multiple-comparison test and Student’s *t* test for 2-group comparisons, when appropriate. A 2-tailed *P* value of <0.05 was considered statistically significant for all tests used in this study. Statistical analyses were performed using Stata software, version 15.1 (Stata Corp.), and Prism 9 (GraphPad software).

### Study approval.

The study was approved by the IRB of Weill Cornell Medicine. The study was carried out in strict accordance with the recommendations in the *Guide for the Care and Use of Laboratory Animals* (National Academies Press, 2011), and all mice were housed in accordance with Weill Cornell Medicine guidelines. All procedures involving mice were approved by the Weill Cornell Medicine IACUC. Studies were conducted in accordance with the Declaration of Helsinki. We received written consent preoperatively from patients with a diagnosis of PNETs for study enrollment between the years 2007 and 2021. Clinical specimens were collected from patients undergoing surgery at Weill Cornell Medicine/New York Presbyterian Hospital. Clinical characteristics of the patients and tumors were collected via chart review.

## Author contributions

JG, JL, XC, BMF, RZ, YCND, OE, TJF, and IMM designed the research study. JG, JL, ZL, KJC, YJL, XC, and IMM conducted experiments. JG, JL, AV, DK, MDM, TMU, ZL, RB, and IMM performed data analysis. JG, JL, AV, DK, MDM, TMU, JWT, DS, RB, RZ, BMF, TS, TJF, CEE, and IMM were involved in data acquisition. JG, JL, AV, DK, XC, YCND, and IMM wrote the manuscript. OE, TJF, and IMM were involved in conceptualization and securing funding. OE, TJF, and IMM supervised the study. All authors participated in interpreting the results and revising the manuscript. The order of the co–first authors was determined by their relative contributions to the study.

## Supplementary Material

Supplemental data

Supplemental table 1

Supplemental table 2

## Figures and Tables

**Table 1:**
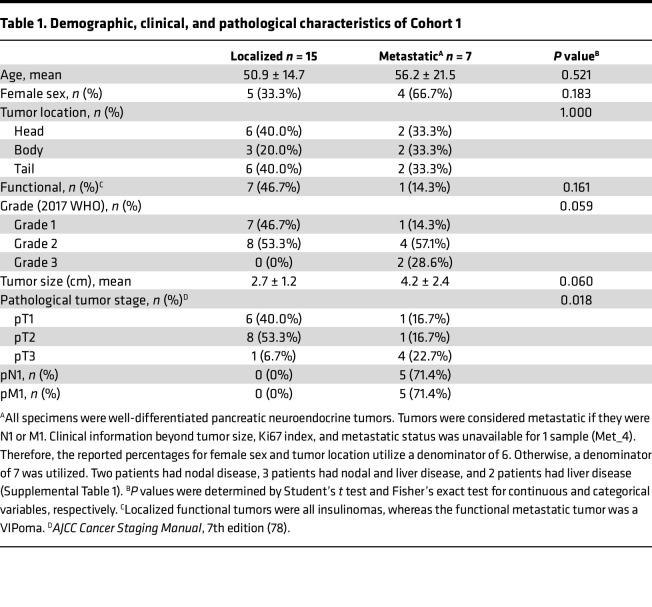
Demographic, clinical, and pathological characteristics of Cohort 1

## Version 1 10/27/2022

In-Press Preview

## Version 2 12/08/2022

Electronic publication
