# Supplementary material for: Metastatic pancreatic neuroendocrine tumors feature elevated T cell infiltration
Source: JCI Insight. 2022 Dec 8;7(23):e160130. doi: 10.1172/jci.insight.160130 (PMC9746918; doi:10.1172/jci.insight.160130)
Supplement: Supplemental data [file jciinsight-7-160130-s087.pdf]

SUPPLEMENTARY FIGURES

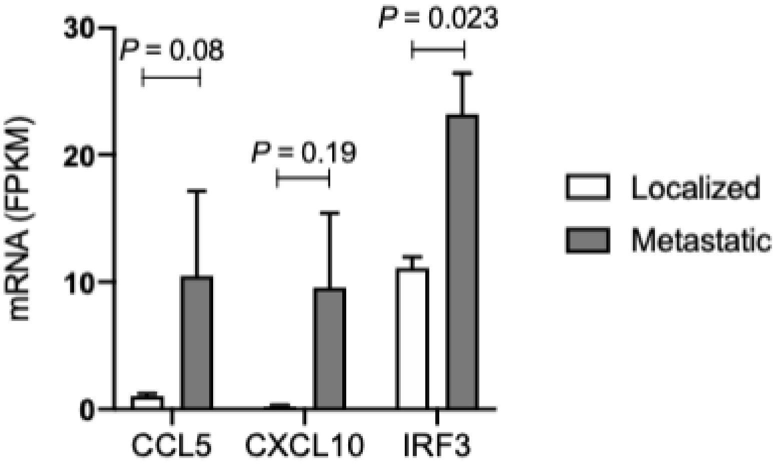

Supplementary Figure 1. RNA expression of chemokines in PNETs.

RNA expression as measured by normalized FPKM of *CCL5*, *CXCL10*, and *IRF3* between localized and metastatic PNETs. FDR adjusted *P*-values are indicated on the graph. Data represents mean ± SEM.

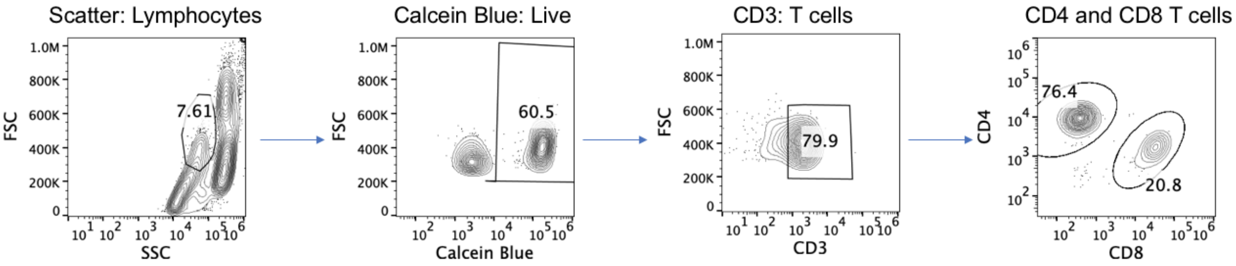

Supplementary Figure 2. Gating strategy utilized for selection of tumor infiltrating T cells.

Representative gating strategy utilized to identify tumor-infiltrating T lymphocytes. Utilizing sizing derived from PBMC results, lymphocyte populations were first selected based on forward-scatter (FSC) and side-scatter (SSC). Live cells were identified by selecting for those staining positive for Calcein Blue, AM. Next, T cells were identified by selecting the $CD3^+$ population, from which both $CD4^+$ and $CD8^+$ T cells were derived.

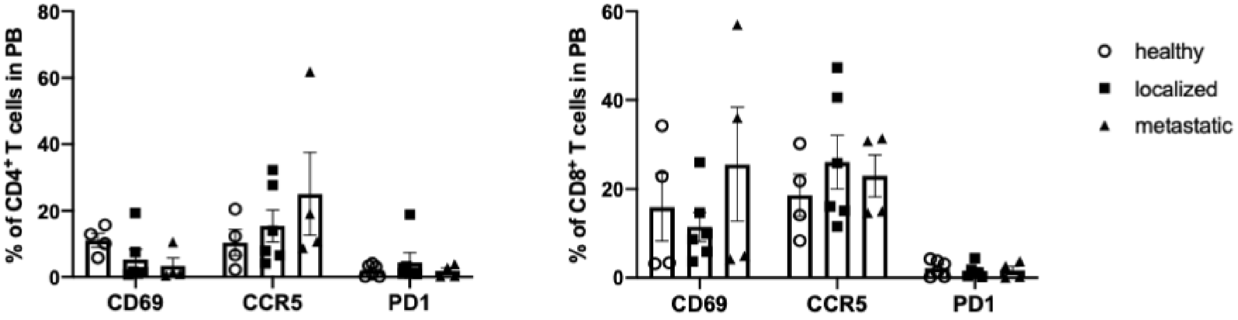

Supplementary Figure 3. Summary data of peripheral T cell phenotype analysis in PNET patients and healthy donors.

Summary data of flow cytometry frequency analysis of $CD69^+$, $CCR5^+$, and $PD1^+$ among $CD4^+$ and $CD8^+$ T cells in peripheral blood of healthy controls and PNET patients. *P* value did not reach statistical significance when analyzed by Kruskal-Wallis test with Dunn's multiple comparisons.

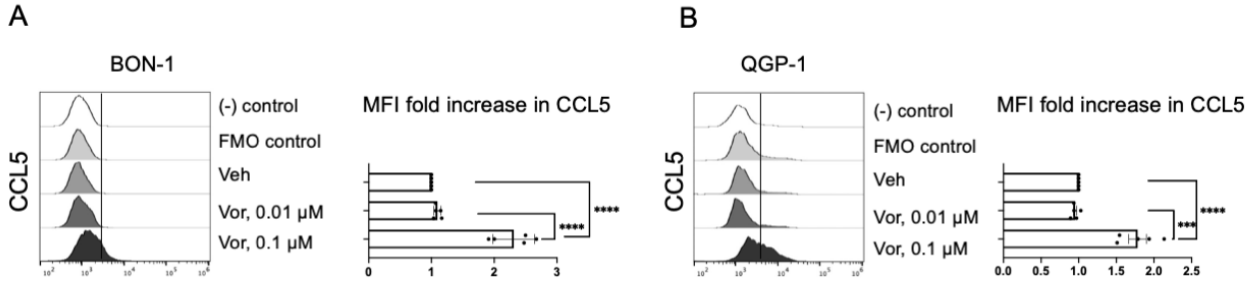

Supplementary Figure 4. Measurement of CCL5 expression by flow cytometry analysis.

BON-1 (A) and QGP-1 (B) cells were treated with subtoxic doses of vorinostat (vor) for 72 hr. (0.01 μM and 0.1 μM). Representative flow cytometry plots are shown on the left. Fold increase of mean fluorescence intensity (MFI) was obtained by normalization of CCR5 expression in vorinostat-treated cells by DMSO-treated cells. *P* value was determined by one-way ANOVA test (***, $P < 0.001$; ****, $P < 0.0001$). Experiments were repeated at least 2 times independently (n = 4-5).

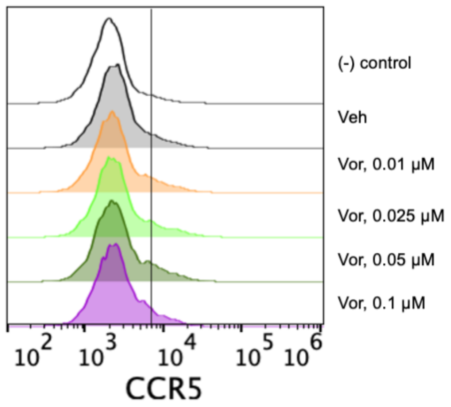

Supplementary Figure 5. CCR5 expression on T cells are not increased following vorinostat treatment.

Representative flow cytometry plots of CCR5 expression changes on $CD3^+$ T cells following 72 hr. treatment (0.01 µM, 0.025 µM, 0.05 µM, and 0.1 µM) with vorinostat. Cells gated on live $CD3^+$ T cells isolated from peripheral blood. No appreciable changes to T cell CCR5 expression were identified following drug treatment.

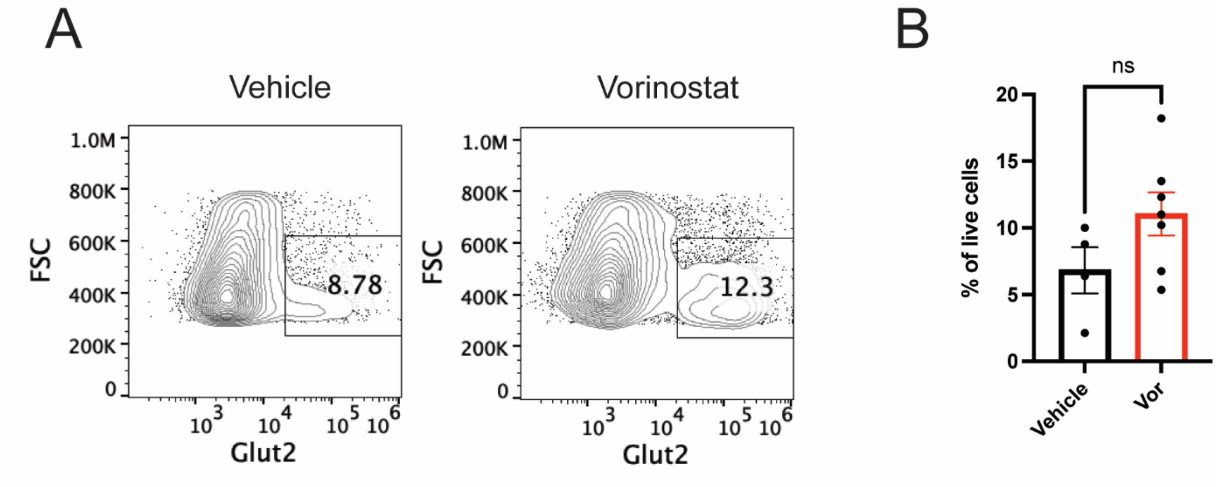

Supplementary Figure 6. Analyses of SLC2A2+ (Glut2+) expressing tumor cells in a mouse PNET model.

(A) Representative flow cytometry plots showing Glut2 expression in gated live cells isolated from subcutaneous mouse PNET. (B) Frequencies of $Glut2^{+}$ PNETs isolated from mice either treated with vehicle control or vorinostat are plotted. *P* value was determined by Mann-Whitney test (ns, not significant). (n = 4 and 7 for vehicle and vorinostat treatment groups, respectively).

Supplementary Table

Supplementary Table 2. Demographics, clinical, and pathological characteristics of patients evaluated by immunohistochemistry.

	Localized n = 9	Metastatic n = 9	*P*-value[c]
Age, mean	55.1 ± (17.7)	59.0 ± (20.0)	0.758
Female sex, n (%)	2 (22.2%)	7 (77.8%)	0.154
Tumor location, n (%)			
Head	4 (44.4%)	3 (33.3%)	0.788
Body	1 (11.1%)	2 (22.2%)	
Tail	4 (44.4%)	4 (44.4%)	
Functional, n (%)[a]	4 (44.4%)	1 (12.5%)	0.294
Grade (2017 WHO), n (%)			
Grade 1	5 (55.6%)	1 (12.5%)	0.094
Grade 2	4 (44.4%)	5(62.5%)	
Grade 3	0	2 (25%)	
Tumor size (cm), mean	2.0 ± 0.9	4.0 ± 2.3	0.028
Pathological tumor stage, n (%)[d]			
pT1	6 (66.6%)	2(25%)	0.207
pT2	2 (22.2%)	3 (37.5%)	
pT3	1 (11.1%)	3 (37.5%)	
pN1, n (%)	0 (0%)	8 (88.9%)	
pM1, n (%)	0 (0%)	7 (77.9%)	

[a]Localized functional tumors were all insulinomas, whereas the functional metastatic tumor was a VIPoma.
[b]All specimens were well-differentiated pancreatic neuroendocrine tumors. Tumors were considered metastatic if they were N1 or M1. Clinical information was limited for one patient in the

metastatic cohort. Therefore, the reported percentages for functional status, grade, and pT stage for the metastatic group utilize a denominator of 8. Otherwise, a denominator of 9 was utilized.
[c]*P*-values were determined by Student's *t* test and Fisher's exact test for continuous and categorical variables, respectively.
[d] AJCC Cancer Staging Manual, 7th edition.
