# Supplementary material for: Metastatic pancreatic neuroendocrine tumors feature elevated T cell infiltration
Source: JCI Insight. 2022 Dec 8;7(23):e160130. doi: 10.1172/jci.insight.160130 (PMC9746918; doi:10.1172/jci.insight.160130)
Supplement: Supplemental table 2 [file jciinsight-7-160130-s089.pdf]

Supplementary Table

Supplementary Table 2. Demographics, clinical, and pathological characteristics of patients evaluated by immunohistochemistry.

	Localized n = 9	Metastatic n = 9	*P*-value[c]
Age, mean	55.1 ± (17.7)	59.0 ± (20.0)	0.758
Female sex, n (%)	2 (22.2%)	7 (77.8%)	0.154
Tumor location, n (%)			
Head	4 (44.4%)	3 (33.3%)	0.788
Body	1 (11.1%)	2 (22.2%)	
Tail	4 (44.4%)	4 (44.4%)	
Functional, n (%)[a]	4 (44.4%)	1 (12.5%)	0.294
Grade (2017 WHO), n (%)			
Grade 1	5 (55.6%)	1 (12.5%)	0.094
Grade 2	4 (44.4%)	5(62.5%)	
Grade 3	0	2 (25%)	
Tumor size (cm), mean	2.0 ± 0.9	4.0 ± 2.3	0.028
Pathological tumor stage, n (%)[d]			
pT1	6 (66.6%)	2(25%)	0.207
pT2	2 (22.2%)	3 (37.5%)	
pT3	1 (11.1%)	3 (37.5%)	
pN1, n (%)	0 (0%)	8 (88.9%)	
pM1, n (%)	0 (0%)	7 (77.9%)	

[a]Localized functional tumors were all insulinomas, whereas the functional metastatic tumor was a VIPoma.
[b]All specimens were well-differentiated pancreatic neuroendocrine tumors. Tumors were considered metastatic if they were N1 or M1. Clinical information was limited for one patient in the

metastatic cohort. Therefore, the reported percentages for functional status, grade, and pT stage for the metastatic group utilize a denominator of 8. Otherwise, a denominator of 9 was utilized.
[c]*P*-values were determined by Student's *t* test and Fisher's exact test for continuous and categorical variables, respectively.
[d] AJCC Cancer Staging Manual, 7th edition.
